# Chronic use of non-steroidal anti-inflammatory drugs (NSAIDs) or acetaminophen and relationship with mortality among United States Veterans after testing positive for COVID-19

**DOI:** 10.1371/journal.pone.0267462

**Published:** 2022-05-05

**Authors:** Heather M. Campbell, Allison E. Murata, Todd A. Conner, Greg Fotieo

**Affiliations:** 1 Cooperative Studies Program, Clinical Research Pharmacy Coordinating Center, US Department of Veterans Affairs, Albuquerque, New Mexico, United States of America; 2 New Mexico VA Healthcare System, US Department of Veterans Affairs, Albuquerque, New Mexico, United States of America; Azienda Ospedaliero Universitaria Careggi, ITALY

## Abstract

Non-steroidal anti-inflammatory drugs (NSAIDs) and acetaminophen are among the most-frequently used medications. Although these medications have different mechanisms of action, they have similar indications and treatment duration has been positively correlated with cardiovascular risk although the degree of risk varies by medication. Our objective was to study treatment effects of chronic use of individual NSAID medications and acetaminophen on all-cause mortality among patients who tested positive for COVID-19 while accounting for adherence. We used the VA national datasets in this retrospective cohort study to differentiate between sporadic and chronic medication use: sporadic users filled an NSAID within the last year, but not recently or regularly. Using established and possible risk factors for severe COVID-19, we used propensity scores analysis to adjust for differences in baseline characteristics between treatment groups. Then, we used multivariate logistic regression incorporating inverse propensity score weighting to assess mortality. The cohort consisted of 28,856 patients. Chronic use of aspirin, ibuprofen, naproxen, meloxicam, celecoxib, diclofenac or acetaminophen was not associated with significant differences in mortality at 30 days (OR = 0.98, 95% CI: 0.95–1.00; OR = 0.99, 95% CI: 0.98–1.00; OR = 1.00, 95% CI: 0.98–1.01; OR = 0.99, 95% CI: 0.98–1.00; OR = 1.00, 95% CI: 0.98–1.01; OR = 0.99, 95% CI: 0.97–1.01; and OR = 1.00, 95% CI: 0.99–1.02, respectively) nor at 60 days (OR = 0.97, 95% CI: 0.95–1.00; OR = 1.00, 95% CI: 0.99–1.01; OR = 0.99, 95% CI: 0.98–1.01; OR = 0.99, 95% CI: 0.97–1.00; OR = 0.99, 95% CI: 0.97–1.01; OR = 0.99, 95% CI: 0.97–1.01; and OR = 1.01, 95% CI: 0.99–1.02, respectively). Although the study design cannot determine causality, the study should assure patients as it finds no association between mortality and chronic use of these medications compared with sporadic NSAID use among those infected with COVID-19.

## Introduction

The emergence of the COVID-19 virus presented an immediate challenge to the scientific community. Because of the novel nature of COVID-19, there was no established understanding of risk or protective factors for severity of disease. The scientific community began to search for potential risk and protective factors. NSAIDs were identified as a potential candidate therapeutic class due to their mechanism of action. Authors of an early correspondence suggested ibuprofen’s ability to stimulate production of ACE2, an enzyme by which SARS-CoV-2 binds to its target cell, places patients at increased risk for worse COVID-19 outcomes [1]. Contradictorily, another correspondence suggested ibuprofen might prevent COVID-19 fatalities by reducing cytokine release syndrome found in severely ill COVID-19 patients by reducing interleukin-6 levels [2–4]. Subsequently, the European Medications Agency stated there was no evidence to suggest ibuprofen or non-steroidal anti-inflammatory drugs (NSAIDs) put patients at risk: rather they recommended that clinicians perform a risk assessment reminding them that paracetamol (i.e., acetaminophen) is the first-line treatment for fever [5]. The U.S. Federal Drug Administration also stated there was no evidence of NSAIDs putting patients at risk, though their communication did not specify acetaminophen as a first-line treatment [6].

According to the Centers for Disease Control and Prevention, history of cardiovascular disease, history of systemic inflammatory disorder, older age, type 2 diabetes mellitus, and smoking are a subset of established risk factors of severe COVID-19; hypertension and type 1 diabetes mellitus are possible risk factors, among others [7, 8]. Unrelated to COVID-19, these factors also increase risk of cardiovascular events with NSAID use [9–12]. There is further evidence that cardiovascular risk is increased as NSAID exposure is increased and that cardiovascular risk varies by NSAID, potentially due to cyclooxygenase (COX) inhibition selectivity [13]. Similar evidence exists for acetaminophen use and duration of therapy increasing cardiovascular risk [13]. The investigators of a meta-analysis found patients with a higher risk of vascular disease who took high-dose diclofenac or COX-2 inhibitors had higher risk of major cardiovascular events compared to placebo while those taking high-dose naproxen did not [9]. Additionally, patients taking diclofenac, COX-2 inhibitors, or naproxen were at higher risk for major coronary events. Our objective was to study effects of chronic use of individual NSAID medications and acetaminophen on all-cause mortality among patients who tested positive for COVID-19 while accounting for adherence.

## Materials and methods

This article follows the Strengthening the Reporting of Observational Studies in Epidemiology guideline. The New Mexico Veterans Affairs Health Care System’s Institutional Review Board approved this study (20-H319/H3234). Consent was not obtained; they approved a waiver as, among other things, the research could not be carried out practically with the number of patients in this retrospective database analysis. We used the national databases at the VA Corporate Data Warehouse (CDW) and the VA COVID-19 Shared Data Resource to extract data for this study. If a Veteran who received VA healthcare services died or reported testing positive outside the VA, this was also captured. The VA CDW has about 94% accuracy capturing date of death; when looking at month and year of death only, this increases to 99% [14].

We incorporated a quasi-experimental study design. We followed patients who tested positive for COVID-19 from March 2, 2020 to December 14, 2020 for 30- and 60- day all-cause mortality from time of diagnosis. December 14, 2020 was the end date to eliminate confounding associated with distribution of the first U.S. COVID-19 vaccine for clinical use. We selected 30- and 60- day mortality to assess robustness of results.

The VA pharmacy database was queried for the year before testing positive for COVID-19 to classify patients into two patterns of use: sporadic NSAID and chronic NSAID or acetaminophen use. Studied NSAIDs included aspirin > 150mg/day, ibuprofen, naproxen, meloxicam, celecoxib, and diclofenac. To help differentiate between sporadic and chronic use, we wanted to ensure sporadic users did not have an NSAID prescription within the 90 days before the 14-day symptomatic window; doing this also aids in NSAID use not being confounded by COVID-19 severity. We used medication possession ratio (MPR) to measure adherence by evaluating fill dates and days supply of individual prescriptions longitudinally to categorize users. A sporadic user was defined as filling a prescription one or more times over the year resulting in a MPR < 0.75. A chronic user was defined as filling one medication amounting to a MPR ≥ 0.75 with a supply on-hand within the 14 days before testing positive. We used documented sporadic NSAID use as the control rather than no documented use to limit confounding by undocumented NSAID consumption; if patients fill prescriptions for NSAIDs in the VA they may be less likely to buy them over-the-counter.

Since low-dose aspirin does not systemically inhibit COX enzymes, the mechanism of action of NSAIDs, patients taking low-dose aspirin were not considered NSAID users unless they otherwise met the criteria for another NSAID. We also compared chronic acetaminophen use to sporadic NSAID use since acetaminophen can be taken for similar indications as NSAIDs. Comorbidities were captured if ICD-10 code documentation existed for the two years before testing positive.

### Statistical analysis

All analyses were conducted using StataMP 15 (StataCorp LP, College Station, TX). We included age, sex, race and ethnicity, urban/suburban versus rural living, body mass index, smoking status, cardiovascular disease, systemic inflammatory disorder (defined as rheumatoid arthritis, systemic lupus erythematosus, or spondyloarthritis), hypertension, hyperlipidemia, type 2 diabetes mellitus, cancer, chronic kidney disease, chronic obstructive pulmonary disease, immunocompromised status (categorized as due to solid organ transplant or not), sickle cell disease, asthma, cerebrovascular disease, liver disease, dementia/Alzheimer’s disease, type 1 diabetes mellitus, and number of Elixhauser comorbidities as covariates in the study. Sex, race, and ethnicity were preferentially defined based on patient declaration; if data were missing the algorithm based these categories on majority declared by VA staff. We also incorporated test month and test season to account for the changing clinical management, weather, and population health behaviors during the study timeframe.

We used propensity scores analysis to adjust for differences in baseline characteristics between treatment groups. If confounders across groups become similar after such adjustment researchers can obtain an unbiased estimate of the treatment effect [15]. Through an iterative process of modeling the propensity score, one can attempt to achieve balance. If any covariates remain unbalanced, a second step is used for further adjustment, though bias will still exist [15]. A standardized mean difference > 0.1 is considered important. We used two-stage propensity scores analysis: in the first stage, the regression model predicts the treatment selection; in the second stage, the regression model predicts the outcome by using the inverse of the weight of the propensity score. Covariates related to the outcome should be used in both stages [16]. This method is considered doubly-robust, meaning even if one of the two models is mis-specified the estimator is still consistent. We started by including all covariates mentioned above, then selected the model with the best balance based on established approaches.

#### Additional analyses

We stratified analyses of treatment effects for covariates with odds ratios (ORs) ≥ 2.0 to evaluate distinct effects among those having, and not having, the characteristic. Due to differences in their mechanisms of action, we also compared chronic NSAID medication use to chronic acetaminophen use.

## Results

**Fig 1:**
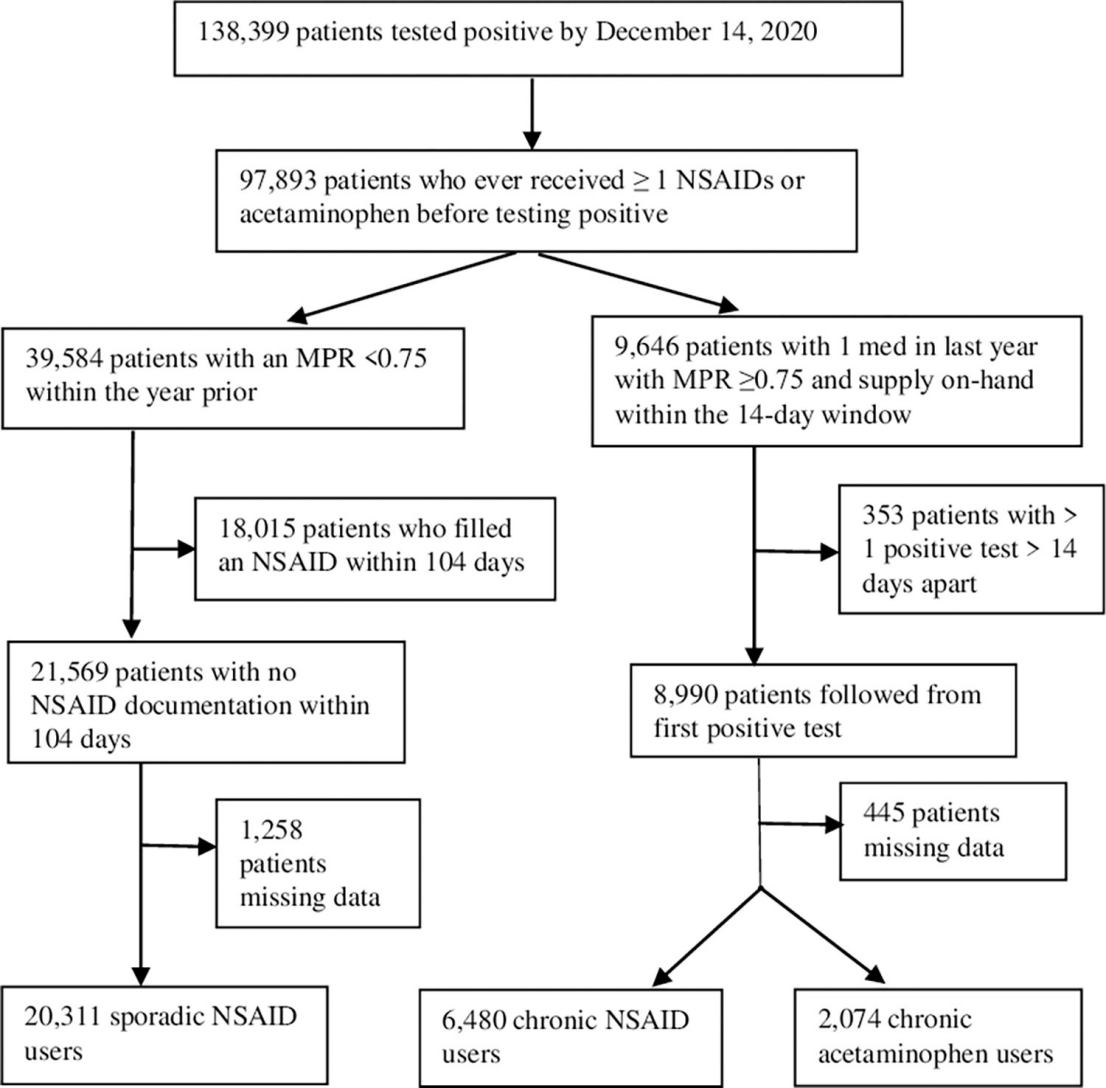
Patients testing positive who had documented NSAID or acetaminophen use. NSAID, non-steroidal anti-inflammatory drug; MPR, medication possession ratio.

Our cohort consisted of 28,856 patients: 20,311 sporadic NSAID users, 6,480 chronic NSAID monotherapy users, and 2,074 chronic acetaminophen users (Fig 1). The mean ± standard deviation age of patients who tested positive for COVID-19 and used NSAIDs was 57.88 ± 15.39 years. These patients were 87.40% male. Whites, Blacks, and American Indians/Alaska Natives comprised 60.22%, 29.83%, and 1.03% of this group, respectively (Table 1). For acetaminophen, age was 67.10 ± 14.36 years, 93.15% were male, and 56.36%, 34.19%, and 1.11% were White, Black, and American Indian/Alaska Native, respectively. At 30 days from testing positive for COVID-19, all-cause mortality was 4.67% for sporadic users, 3.01% for chronic NSAID users, and 8.68% for chronic acetaminophen users. By 60 days, 5.54% of sporadic users, 3.57% of chronic NSAID users, and 10.56% of chronic acetaminophen users had died from any cause. Among chronic NSAID users, 30-day mortality was 7.64% for aspirin, 2.54% for ibuprofen, 2.99% for naproxen, 2.53% for meloxicam, 3.10% for celecoxib, and 2.89% for diclofenac. At 60 days, mortality was 8.83%, 3.25%, 3.26%, 3.06%, 3.36%, and 3.37%, respectively. Across treatment groups, there were substantial differences in baseline characteristics before propensity score weighting. Table 2 reveals unadjusted ORs of the baseline characteristics.

**Table 1:** Baseline characteristics by treatment group.

	Aspirin (N = 419)	Ibuprofen (N = 1,814)	Naproxen (N = 1,136)	Meloxicam (N = 2,092)	Celecoxib (N = 387)	Diclofenac (N = 623)	Sporadic NSAID use (N = 20,311)	Acetaminophen (N = 2,074)	Total (N = 28,856)
**Demographics**									
Age (mean ± standard deviation)	69.56 ± 10.52	54.74 ± 13.36	56.75 ± 13.36	58.46 ± 13.34	57.18 ± 13.78	57.21 ± 12.58	57.96 ± 15.93	67.10 ± 14.36	58.54 ± 15.50
Male (N (%))	407 (97.14)	1,557 (85.83)	992 (87.32)	1,817 (86.85)	326 (84.24)	534 (85.71)	17,774 (87.51)	1,932 (93.15)	25,341 (87.82)
Urban/suburban living (N (%))	322 (76.85)	1,508 (83.13)	904 (79.58)	1,605 (76.72)	306 (79.07)	481 (77.21)	17,195 (84.66)	174 (83.75)	24,057 (83.37)
Race									
White (N (%))	267 (63.72)	1,038 (57.22)	698 (61.44)	1,392 (66.54)	268 (69.25)	402 (64.53)	12,062 (59.39)	1,136 (56.36)	17,296 (59.94)
Black (N (%))	127 (30.31)	570 (31.42)	337 (29.67)	525 (25.10)	82 (21.19)	183 (29.37)	6,166 (30.36)	709 (34.19)	8,699 (30.15)
Asian (N (%))	1 (0.24)	18 (0.99)	6 (0.53)	11 (0.53)	5 (1.29)	4 (0.64)	217 (1.07)	15 (0.72)	277 (0.96)
American Indian/Alaska Native (N (%))	3 (0.72)	22 (1.21)	12 (1.06)	17 (0.81)	8 (2.07)	3 (0.48)	212 (1.04)	23 (1.11)	300 (1.04)
Native Hawaiian/Pacific Islander (N (%))	2 (0.48)	18 (0.99)	7 (0.62)	18 (0.86)	0 (0.00)	2 (0.32)	223 (1.10)	22 (1.06)	292 (1.01)
Unknown (N (%))	19 (4.53)	148 (8.16)	76 (6.69)	129 (6.17)	24 (6.20)	29 (4.65)	1,431 (7.05)	136 (6.56)	1,992 (6.90)
Ethnicity									
Hispanic/Latino (N (%))	16 (3.82)	234 (12.90)	131 (11.53)	205 (9.80)	38 (9.82)	49 (7.87)	2,590 (12.75)	219 (2.56)	3,482 (12.07)
Not Hispanic/Latino (N (%))	392 (93.56)	1,526 (84.12)	964 (84.86)	1,827 (87.33)	341 (88.11)	553 (88.76)	17,147 (84.42)	1,800 (86.79)	24,550 (85.08)
Unknown (N (%))	11 (2.63)	54 (2.98)	41 (3.61)	60 (2.87)	8 (2.07)	21 (3.37)	574 (2.83)	55 (2.65)	824 (2.86)
Season of COVID test									
Spring (N (%))	78 (18.62)	261 (14.39)	163 (14.35)	212 (10.13)	38 (9.82)	58 (9.31)	2,602 (12.81)	458 (22.08)	3,870 (13.41)
Summer (N (%))	133 (31.74)	722 (39.80)	420 (36.97)	766 (36.62)	140 (36.18)	248 (39.81)	6,249 (30.77)	798 (38.48)	9,476 (32.84)
Fall (N (%))	208 (49.64)	831 (45.81)	553 (48.68)	1,114 (53.25)	209 (54.01)	317 (50.88)	11,460 (56.42)	818 (39.44)	15,510 (53.75)
Smoking status									
Never (N (%))	124 (29.59)	798 (43.99)	495 (43.57)	887 (42.40)	158 (40.83)	280 (44.94)	8,451 (41.61)	800 (38.57)	11,993 (41.56)
Former (N (%))	240 (57.28)	711 (39.20)	461 (40.58)	908 (43.40)	174 (44.96)	255 (40.93)	8,334 (41.03)	986 (47.54)	12,069 (41.82)
Current (N (%))	46 (10.98)	271 (14.94)	145 (12.76)	244 (11.66)	42 (10.85)	75 (12.04)	2,795 (13.76)	223 (10.75)	3,841 (13.31)
Unknown (N (%))	9 (2.15)	34 (1.87)	35 (3.08)	53 (2.53)	13 (3.36)	13 (2.09)	731 (3.60)	65 (3.13)	953 (3.30)
Body mass index									
Normal weight (N (%))	59 (14.08)	159 (8.77)	87 (7.66)	188 (8.99)	30 (7.75)	38 (6.10)	2,746 (13.52)	306 (14.75)	3,613 (12.52)
Overweight (N (%))	149 (35.56)	516 (28.45)	330 (29.05)	573 (27.39)	121 (31.27)	165 (26.48)	6,202 (30.54)	638 (30.76)	8,694 (30.13)
Obese (N (%))	211 (50.36)	1,139 (62.79)	719 (63.29)	1,331 (63.62)	236 (60.98)	420 (67.42)	11,363 (55.95)	1,130 (54.48)	16,549 (57.35)
**Comorbidities**									
Cardiovascular disease (N (%))	241 (57.52)	256 (14.11)	173 (15.23)	381 (18.21)	72 (18.60)	114 (18.30)	4,919 (24.22)	80 (38.72)	6,960 (24.12)
Systemic inflammatory disorder (N (%))	79 (18.85)	323 (17.81)	240 (21.13)	498 (23.80)	116 (29.97)	160 (25.68)	3,508 (17.27)	51 (24.54)	54.34 (18.83)
Hypertension (N (%))	367 (87.59)	1,101 (60.69)	743 (65.40)	1,436 (68.64)	251 (64.86)	415 (66.61)	13,060 (64.30)	171 (82.64)	19,088 (66.15)
Hyperlipidemia (N (%))	340 (81.15)	1,098 (60.53)	713 (62.76)	1,395 (66.68)	253 (65.37)	397 (63.72)	12,508 (61.58)	151 (72.57)	18,208 (63.10)
Type 2 diabetes mellitus (N (%))	240 (57.28)	569 (31.37)	352 (30.99)	715 (34.18)	128 (33.07)	228 (36.60)	7,135 (35.13)	102 (48.94)	10,382 (35.98)
Cancer (N (%))	49 (11.69)	110 (6.06)	67 (5.90)	150 (7.17)	19 (4.91)	34 (5.46)	1,639 (8.07)	24 (11.72)	2,311 (8.01)
Chronic kidney disease (N (%))	111 (26.49)	63 (3.47)	52 (4.58)	94 (4.49)	17 (4.39)	26 (4.17)	2,527 (12.44)	51 (24.83)	3,405 (11.80)
Chronic obstructive pulmonary disease (N (%))	96 (22.91)	211 (11.63)	129 (11.36)	249 (11.90)	61 (15.76)	77 (12.36)	2,801 (13.79)	52 (25.02)	4,144 (14.36)
Immunocompromised from solid organ transplant (N (%))	6 (1.43)	1 (0.06)	0 (0.00)	2 (0.10)	0 (0.00)	0 (0.00)	116 (0.57)	2 (0.82)	141 (0.49)
Sickle cell disease (N (%))	1 (0.24)	3 (0.17)	1 (0.09)	3 (0.14)	0 (0.00)	1 (0.16)	51 (0.25)	1 (0.53)	69 (0.24)
Asthma (N (%))	27 (6.44)	176 (9.70)	118 (10.39)	213 (10.18)	37 (9.56)	58 (9.31)	1,781 (8.77)	20 (9.79)	2,614 (9.06)
Cerebrovascular disease (N (%))	144 (34.37)	67 (3.69)	59 (5.19)	123 (5.88)	15 (3.88)	29 (4.65)	1,932 (9.51)	37 (17.74)	2,738 (9.49)
Immunocompromised other than solid organ transplant (N (%))	76 (18.14)	239 (13.18)	152 (13.38)	305 (14.58)	50 (12.92)	89 (14.29)	2,779 (13.68)	43 (20.59)	4,115 (14.26)
Liver disease (N (%))	35 (8.35)	179 (9.87)	107 (9.42)	187 (8.94)	35 (9.04)	48 (7.70)	2,001 (9.85)	21 (10.27)	2,805 (9.72)
Dementia/Alzheimer’s disease (N (%))	43 (10.26)	29 (1.60)	28 (2.46)	43 (2.06)	7 (1.81)	13 (2.09)	1,074 (5.29)	27 (13.16)	1,509 (5.23)
Type 1 diabetes mellitus (N (%))	17 (4.06)	25 (1.38)	13 (1.14)	46 (2.20)	6 (1.55)	13 (2.09)	459 (2.26)	8 (3.95)	661 (2.29)
Number of Elixhauser comorbidities (mean ± standard deviation)	8.42 ± 8.48	3.38 ± 5.65	3.21 ± 5.29	3.55 ± 5.74	3.45 ± 5.76	3.25 ± 5.41	5.34 ± 7.39	9.15 ± 8.83	5.25 ± 7.39

**Table 2:** Unadjusted odds ratios and p-values associated with 30-day and 60-day mortality.

	30-day mortality		60-day mortality	
	Odds ratio	P-value	Odds ratio	P-value
Test month	0.90	<0.001	0.91	<0.001
Test season	0.66	<0.001	0.68	<0.001
Age (in years)	1.10	<0.001	1.10	<0.001
Age (< 65 or ≥ 65 years)	10.94	<0.001	10.29	<0.001
Male	4.89	<0.001	4.54	<0.001
Urban/suburban versus rural	0.85	0.020	0.80	0.001
Race (White, Black, Asian, American Indian/Alaska Native, Native Hawaiian/Pacific Islander, Unknown)	1.01	0.595	1.00	0.966
Race (White, Black, Other)	0.98	0.715	0.95	0.236
Ethnicity (Hispanic/Latino, Not Hispanic/Latino, Other)	0.98	0.435	0.99	0.429
Ethnicity (Hispanic/Latino, Other)	0.81	0.026	0.83	0.025
Smoking status (never, former, current, unknown)	1.16	<0.001	1.16	<0.001
Body mass index (normal weight, overweight, obese)	0.69	<0.001	0.67	<0.001
Cardiovascular disease	3.19	<0.001	3.23	<0.001
Systemic inflammatory disorder	0.92	0.241	0.95	0.437
Hypertension	3.96	<0.001	4.00	<0.001
Hyperlipidemia	1.83	<0.001	1.84	<0.001
Type 2 diabetes mellitus	2.62	<0.001	2.64	<0.001
Cancer	1.80	<0.001	1.85	<0.001
Chronic kidney disease	3.68	<0.001	3.68	<0.001
Chronic obstructive pulmonary disease	2.44	<0.001	2.52	<0.001
Immunocompromised from solid organ transplant	2.47	<0.001	2.37	<0.001
Sickle cell disease	0.30	0.169	0.25	0.233
Asthma	0.69	0.001	0.67	<0.001
Cerebrovascular disease	3.19	<0.001	3.25	<0.001
Immunocompromised other than solid organ transplant	1.53	<0.001	1.56	<0.001
Liver disease	0.99	0.947	0.94	0.473
Dementia/Alzheimer’s disease	7.45	<0.001	7.39	<0.001
Type 1 diabetes mellitus	2.00	<0.001	2.13	<0.001
Number of Elixhauser comorbidities	1.08	<0.001	1.08	<0.001
Number of Elixhauser comorbidities (<10, 10–19, ≥20)	2.29	<0.001	2.31	<0.001

Our final model comparing chronic to sporadic use included covariates with ORs ≥ 2.0, denoting a moderate association with mortality [17]; the only exception being immunocompromised due to solid organ transplant as the number of patients was too small: three groups each had zero patients. This approach resulted in very similar baseline covariates: all standardized mean differences were ≤ 0.01 (Table 3); we also had good overlap in propensity scores among the treatment groups. When comparing chronic use of aspirin, ibuprofen, naproxen, meloxicam, celecoxib, diclofenac, and acetaminophen to sporadic NSAID use, we found no significant difference in all-cause mortality at 30 or 60 days apart from meloxicam (OR = 0.99 [95% CI: 0.98–1.00] and OR = 0.99 [95% CI: 0.98–1.00], respectively, p = 0.005 for both). See Table 4.

**Table 3:** Standard mean differences before and after propensity score weighting.

	Aspirin^a^ (N = 419)	Ibuprofen (N = 1,814)	Naproxen (N = 1,136)	Meloxicam (N = 2,092)	Celecoxib (N = 387)	Diclofenac (N = 623)	Acetaminophen (N = 2,074)
Age (< 65 or ≥ 65 years)	0.79; -0.0^b^	-0.29; 0.00	-0.17; 0.00	-0.05; 0.00	-0.13; -0.00	-0.18; -0.00	0.47; -0.01
Male	0.37; -0.00	-0.05; 0.00	-0.01; 0.00	-0.02; 0.00	-0.09; 0.00	-0.05; 0.00	0.19; -0.00
Cardiovascular disease	0.72; -0.01	-0.26; 0.00	-0.23; 0.00	-0.15; 0.00	-0.14; 0.00	-0.15; 0.00	0.32; -0.01
Hypertension	0.57; -0.00	-0.07; 0.00	0.02; 0.00	0.09; 0.00	0.01; -0.00	0.05; 0.00	0.42; -0.00
Type 2 diabetes mellitus	0.46; -0.00	-0.08; 0.00	-0.09; 0.00	-0.02; -0.00	-0.04; 0.00	0.03; 0.00	0.28; -0.01
Chronic kidney disease	0.36; -0.01	-0.34; 0.00	-0.28; 0.00	-0.29; 0.00	-0.29; -0.00	-0.30; 0.00	0.32; -0.01
Chronic obstructive pulmonary disease	0.24; -0.00	-0.06; 0.00	-0.07; -0.00	-0.06; -0.00	0.06; -0.00	-0.04; 0.00	0.29; -0.01
Cerebrovascular disease	0.63; -0.01	-0.24; 0.00	-0.17; -0.00	-0.14; -0.00	-0.23; 0.00	-0.19; 0.00	0.24; -0.00
Dementia/Alzheimer’s disease	0.19; -0.01	-0.20; 0.00	-0.15; 0.00	-0.17; 0.00	-0.19; 0.00	-0.17; 0.00	0.27; -0.01
Type 1 diabetes mellitus	0.10; -0.00	-0.07; -0.00	-0.09; 0.00	-0.00; 0.00	-0.05; -0.00	-0.01; -0.00	0.10; -0.00
Number of Elixhauser comorbidities (< 10, 10–19, ≥ 20)	0.31; -0.00	-0.27; 0.00	-0.29; 0.00	-0.24; 0.00	-0.26; 0.00	-0.28; 0.00	0.38; -0.01

^a^Note sporadic NSAID use is the reference across all treatment groups.

^b^Number before semicolon is the standardized mean difference before propensity score weighting. The number following the semicolon is the standardized mean difference after propensity score weighting.

**Table 4:** Odds ratios of all-cause mortality by treatment group.

	Unadjusted odds ratio	95% confidence interval	P-value	Adjusted odds ratio	95% confidence interval	P-value
	**30-day all-cause mortality**					
Aspirin^a^	1.69	(1.17, 2.43)	0.005	0.98	(0.95, 1.00)	0.103
Ibuprofen	0.53	(0.39, 0.72)	<0.001	0.99	(0.98, 1.00)	0.230
Naproxen	0.63	(0.44, 0.89)	0.009	1.00	(0.98, 1.01)	0.580
Meloxicam	0.53	(0.40, 0.70)	<0.001	0.99	(0.98, 1.00)	0.005
Celecoxib	0.65	(0.37, 1.16)	0.149	1.00	(0.98, 1.01)	0.718
Diclofenac	0.61	(0.38, 0.97)	0.039	0.99	(0.97, 1.01)	0.420
Acetaminophen	1.94	(1.64, 2.29)	<0.001	1.00	(0.99, 1.02)	0.865
	**60-day all-cause mortality**					
Aspirin	1.65	(1.17, 2.33)	0.004	0.97	(0.95, 1.00)	0.061
Ibuprofen	0.57	(0.44, 0.75)	<0.001	1.00	(0.99, 1.01)	0.542
Naproxen	0.57	(0.41, 0.80)	0.001	0.99	(0.98, 1.01)	0.244
Meloxicam	0.54	(0.42, 0.69)	<0.001	0.99	(0.97, 1.00)	0.005
Celecoxib	0.59	(0.34, 1.03)	0.065	0.99	(0.97, 1.01)	0.349
Diclofenac	0.59	(0.38, 0.92)	0.020	0.99	(0.97, 1.01)	0.400
Acetaminophen	2.01	(1.73, 2.34)	<0.001	1.01	(0.99, 1.02)	0.432

^a^Note sporadic NSAID use is the reference across all treatment groups.

Similarly, analyses stratified by age, gender, history of cardiovascular disease, hypertension, type 2 diabetes mellitus, chronic kidney disease, chronic obstructive pulmonary disease, cerebrovascular disease, dementia, or number of comorbidities showed no clinically significant differences though meloxicam was marginally statistically significant at 30 and 60 days. When the comparison group was changed from sporadic NSAID users to chronic acetaminophen users, aspirin and meloxicam were statistically, though not clinically, significant for all-cause mortality at 30 and 60 days.

## Discussion

We found no clinically significant difference comparing chronic use with each of seven medications with sporadic use of an NSAID. This bore out in main and stratified analyses. We also found no clinically significant difference comparing chronic use of individual NSAID medications with chronic acetaminophen use. Our use of MPR rather than simple prescription refill history reflects actual adherence and use patterns more accurately. With our sample size, the analyses had narrow confidence intervals. Due to the standardized mean differences being negligible, we are confident in an unbiased estimate of treatment effect among observed variables with at least a moderate association with all-cause mortality. Further, there was sufficient overlap in propensity scores across treatment groups, also necessary for reduced bias and variance of estimates [18]. Collectively, this study showed that the risk of death in patients who take these medications chronically is no different than patients who sporadically used NSAIDs. This information can also be used by clinicians caring for patients as they make clinical treatment decisions based on their risk assessment. Our attempt to address this was by controlling the month or season in which patients tested positive: with an unadjusted OR = 0.90 and OR = 0.91 for 30-day and 60-day mortality, respectively, month did not meet the criteria for a moderate association to be included in the final propensity score-adjusted model. With season having OR = 0.66 and OR = 0.68 for 30- and 60- day mortality, respectively, it also did not meet the criteria of OR = 0.50 to be included.

At the time of this writing, seventeen studies have focused on potential effects of NSAIDs among COVID-19 patients: three of ibuprofen, one of naproxen, one of celecoxib, and seven of aspirin; the rest kept analysis at the therapeutic class [19–35]. Our study adds compelling evidence to the literature with the second-largest sample size to evaluate chronic NSAID use and the third-largest to evaluate any NSAID use among COVID-19 patients: one study assessed NSAID effects on mortality in the rheumatoid arthritis and osteoarthritis population in the United Kingdom, though adherence was assumed [30]; the other focused on aspirin use at time of testing positive [28]. Three of the seventeen have assessed treatment effects of chronic NSAID use, with only one evaluating a medication effect rather than the therapeutic class and none accounting for adherence: baseline characteristics were not comparable or controlled for in two of the four studies [20, 21, 23, 30]. Among these studies, none found a significant difference in mortality. However, believing this about individual NSAIDs without a more granular assessment can lead to ecological fallacy: what was found for the NSAID class would not necessarily be found for each NSAID medication. In the context of COVID-19 patients, acute use of NSAIDs or acetaminophen was probably used for fever and muscle aches and pains related to the virus in the majority of cases. NSAIDs are taken chronically, however, for osteoarthritis, rheumatoid arthritis, and other chronic musculoskeletal conditions, more prevalent among older adults. Acetaminophen is also used for chronic pain conditions including osteoarthritis and back pain. Therefore, it is clinically important to ascertain possible effects in these patients, especially with the knowledge of associations of increased cardiovascular risk with chronic NSAID or chronic acetaminophen use.

This is the first published study assessing aspirin restricted to a dose > 150mg in which it exhibits the effects of the non-aspirin NSAIDs [36–38]. In the literature, about half studied exclusively low-dose while the other half studied all doses of aspirin. Only one study assessed differential dose effects, separating patients in low- and high- dose aspirin groups [33]. In it, among those taking aspirin, only 51 patients received > 81mg; with mortality rate point estimates of 33.3% and 21.6% for those taking and not taking aspirin, respectively, doubt remained if the authors’ finding of no significance was an artifact of small sample size.

This is also the first study assessing effects of meloxicam or diclofenac among COVID-19 patients for acute or chronic use, before or after testing positive. In our study, 41.90% of chronic NSAID users took these medications. Additionally, we did not identify a study of chronic acetaminophen use among COVID-19 patients. In our study, 24.25% of chronic users were taking acetaminophen. With this study providing evaluation of meloxicam, diclofenac, or acetaminophen in the literature in COVID-19 patients, this is crucial information for providers, as this pertains to 55.99% of chronic users in our study. This also is the first study assessing effects among a documented highly adherent group of patients who tested positive for COVID-19 and who have been taking NSAIDs or acetaminophen chronically.

### Limitations

This study has several limitations. First, since this study is retrospective we can only derive associations from the data; a clinical trial would be needed to establish causal relationships. Also due to its retrospective nature, we were dependent on documentation related to patient characteristics, comorbidities, and mortality; we note the high capture rate of deaths in the CDW. Second, endemic to any propensity scores analysis, we do not know if there are baseline differences between groups for unobserved variables associated with mortality. Having said this, propensity scores analysis reduces the bias of potential unobserved covariates [39]. A specific example of this is our inability to capture potential medications altering the disease course, such as dexamethasone, remdesivir, monoclonal antibody therapy, and experimental administration of COVID-19 vaccines. Third, undoubtedly there was undocumented medication use due to some non-selective NSAIDs’ and acetaminophen’s over-the-counter status in the U.S. The VA may be the ideal healthcare organization to assess NSAID use and effects in the U.S. as many over-the-counter prescriptions are frequently prescribed within the VA: in 2017, about 50% of Veterans had $0 medication copayments while others had a $700/year cap [40]. This means, compared to no usage, having sporadic use as a control reflects a conservative estimate. However, NSAIDs are one of the classes taken most frequently, so this indeed may have more external validity to the general population, serving as an apt control. Fourth, our population has a high proportion of males, though a study has shown similarities between the VA population and the Medicare population [41]. Fifth, although we studied seven different medications, due to small numbers we were not able to look at treatment effects for COVID-19 mortality for every FDA-approved NSAID medication. Lastly, data from other continents is not compared, further limiting generalizability.

## Conclusions

The results of this study show no association between chronic use of any of the six NSAIDs studied or with acetaminophen and all-cause mortality in Veterans diagnosed with COVID-19 infection. Stratified analyses revealed no clinical importance as well, negating the potential of differential effects of chronic use of each medication using the observed covariates in our study. Lastly, a clinically important association was also not revealed when chronic acetaminophen use was substituted for sporadic NSAID use.
